# Paederus Dermatitis Acquired in the Gambia

**DOI:** 10.4269/ajtmh.25-0071

**Published:** 2025-05-27

**Authors:** Olabisi Ogunbiyi, Natasha M. Mawani, Stephen L. Walker

**Affiliations:** ^1^ Hospital for Tropical Diseases, University College London Hospitals NHS Foundation Trust, London, United Kingdom;; ^2^ Department of Cardiology, Conquest Hospital, East Sussex Healthcare NHS Trust, East Sussex, United Kingdom;; ^3^ Department of Dermatology, University College London Hospitals NHS Foundation Trust, London, United Kingdom;; ^4^ Faculty of Infectious and Tropical Diseases, London School of Hygiene and Tropical Medicine, London, United Kingdom

A 28-year-old woman presented with a 4-day history of a sudden-onset painful rash on both arms, diarrhea, and a hoarse voice after recent travel to Senegal and the Gambia. The symptoms started on the second day of her trip to the Gambia. There was no relevant past medical history.

**Figure 1.**
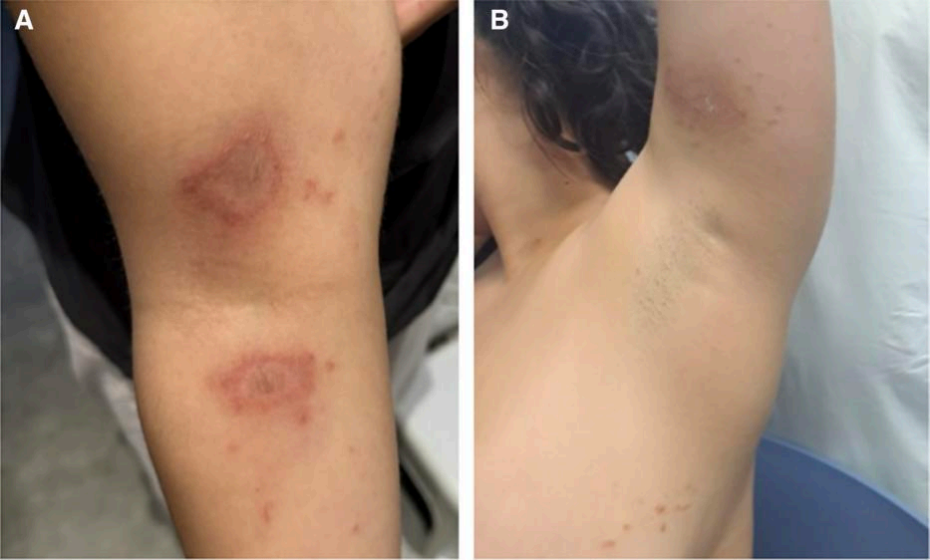
Distribution of lesions (**A**) on the distal right arm and proximal right forearm, as well as those (**B**) on the left arm and breast.

On examination, there were multiple erythematous and hyperpigmented patches with mild peripheral scaling on the right upper limb, the medial aspect of the left arm, and the left breast. The rest of the physical examination was normal. Photographs of early lesions showed central desquamation. The distribution of the lesions on the distal right arm and proximal right forearm (Figure 1A) and those on the left arm and breast (Figure 1B) exhibited the characteristics of “kissing lesions.” These are discrete, well-defined lesions occurring in mirror-image locations on opposing skin surfaces.^1^ This is consistent with a diagnosis of paederus dermatitis, likely acquired in the Gambia.^2^ The rash was treated with clobetasone butyrate 0.05% cream twice daily and emollients.

The diarrhea and hoarse voice resolved spontaneously. *Salmonella* DNA was detected in the stool. A respiratory viral swab result was negative, and HIV and syphilis serology results were also negative. Paederus dermatitis is an acute irritant dermatitis caused by contact with the hemolymph of rove beetles (*Paederus* sp), which contain a vesicant toxin called pederin.^3^ Direct skin contact is not necessary; lesions often appear in areas where the beetle has been crushed or moved. *Paederus* species are widely distributed, and outbreaks have been reported in Europe, Asia, Africa, South America, and Australasia.^4^^,^^5^

A complete skin examination is essential for identifying dermatoses in returned travelers. Appositional patterns should prompt clinicians to consider toxic or irritant dermatitis. There are many other causes of kissing lesions, including candidiasis, impetigo, intertrigo, syphilis, viral warts, molluscum contagiosum, and Lipschütz ulcers.^1^

Paederus dermatitis is a clinical diagnosis based on history and examination. It can present with erythematous or hyperpigmented plaques, as well as ocular involvement (in the form of keratoconjunctivitis and periorbital dermatitis) and genital involvement. Ocular and genital involvement are usually secondary to the transfer of pederin. Kissing lesions are an uncommon manifestation.^6^ Immediate management involves washing the affected area with soap and clean water to remove the toxin. Management includes the use of topical corticosteroids, emollients, cold wet compresses, and antihistamines for symptomatic relief, although data on their effectiveness is lacking.^3^ Preventive approaches that have been advocated include insect-proof mesh and insecticide spraying, but the evidence for these is limited.^6^
